# Stochastic lattice-based modelling of malaria dynamics

**DOI:** 10.1186/s12936-018-2397-z

**Published:** 2018-07-05

**Authors:** Phong V. V. Le, Praveen Kumar, Marilyn O. Ruiz

**Affiliations:** 1 Department of Civil and Environmental Engineering, University of Illinois, Urbana, IL 61801 USA; 2 Faculty of Hydrology, Meteorology and Oceanography, Hanoi University of Science, Vietnam National University, Hanoi, Vietnam; 3 Department of Atmospheric Sciences, University of Illinois, Urbana, IL 61801 USA; 4 Department of Pathobiology, University of Illinois, Urbana, IL 61802 USA

**Keywords:** Malaria, Climate change, Metapopulation, Stochastic, Ecohydrology

## Abstract

**Background:**

The transmission of malaria is highly variable and depends on a range of climatic and anthropogenic factors. In addition, the dispersal of *Anopheles* mosquitoes is a key determinant that affects the persistence and dynamics of malaria. Simple, lumped-population models of malaria prevalence have been insufficient for predicting the complex responses of malaria to environmental changes.

**Methods and results:**

A stochastic lattice-based model that couples a mosquito dispersal and a susceptible-exposed-infected-recovered epidemics model was developed for predicting the dynamics of malaria in heterogeneous environments. The It$\hat{o}$ approximation of stochastic integrals with respect to Brownian motion was used to derive a model of stochastic differential equations. The results show that stochastic equations that capture uncertainties in the life cycle of mosquitoes and interactions among vectors, parasites, and hosts provide a mechanism for the disruptions of malaria. Finally, model simulations for a case study in the rural area of Kilifi county, Kenya are presented.

**Conclusions:**

A stochastic lattice-based integrated malaria model has been developed. The applicability of the model for capturing the climate-driven hydrologic factors and demographic variability on malaria transmission has been demonstrated.

## Background

Malaria is a vector-borne disease with complex nonlinear dynamics [1, 2]. The disease, caused by protozoan parasites of the genus *Plasmodium*, is transmitted between humans by female *Anopheles* mosquitoes. Many factors are determinants for the transmission of malaria, including climate suitability, life cycles of pathogens and vectors, and the local capacity to control the mosquito population [3–6]. The fundamental basis for malaria risk prediction and early warning lies in the ability to estimate the effects of weather, to identify vector habitat, and to model the population dynamics of *Anopheles* mosquitoes and the transmission of the pathogens in the human population.

Mathematical models have been used to provide an explicit framework for understanding malaria transmission dynamics for over 100 years, starting with the pioneering work of Ross [7]. In a simple form, he used a few ordinary differential equations (ODEs) to describe quantitative changes in densities of infected human and mosquitoes. Since then, more sophisticated models have been developed that include factors such as latent periods [4, 8], vector density and human age structure [4], varying human population size and migration [9, 10], socio-economic developments [11], temporary immunity [12], and weather effects [13]. In addition, agent-based and meta-population based models have been used to allow simulations of heterogeneous communities subject to realistic transmission scenarios [14–18]. Over the last 60 years, much scientific research was undertaken and progress made in understanding the biology of malaria vectors and host-parasite-vector interactions. Systematic reviews of mathematical modelling of malaria [19] and other mosquito-borne diseases [20, 21] indicate the need for models to address the complexities is the host-vector-parasite interactions and to incorporate heterogeneous environments.

Deterministic models with susceptible-exposed-infected-recovered (SEIRS) patterns, often based on nonlinear ODEs, are among the standard approaches for estimating the transmission potential for a wide range of infectious diseases, including malaria [22]. These models are useful in understanding the temporal dynamics of infection cycles and in coping with different epidemiological situations including both epidemicity and endemicity. However, SEIRS models ignore the aquatic stage of the mosquito life cycle and the spatial dynamics of mosquitoes when habitats are heterogeneously available, thus limiting the ability to couple and accurately predict the link between the life cycle of *Anopheles* mosquitoes and malaria transmission. Furthermore, deterministic models cannot capture fluctuations dominated by the random nature of population events, environmental conditions, and variability in the controlling parameters, which inevitably occur in a real system [23]. To that end, stochastic models have proved valuable in estimating asymptotic expressions for the probability of occurrence of major outbreaks as well as stochastic extinctions [24, 25]. Nevertheless, there still exists a lack of complete predictive capability which may allow a mechanism to efficiently capture uncertainty in the temporal and spatial dynamics of malaria. This shortcoming has been attributed to the differences in stochastic modelling approaches, as agent-based models [15, 17, 18] are inefficient for large-scale simulations with very large number of vector individuals involved, while lumped-population models [26, 27] bypass the spatial dynamics of the diseases.

In this paper, a stochastic lattice-based integrated malaria (SLIM) model for investigating the dynamics of mosquitoes and transmission of malaria in time and space is developed. More precisely, an entomological mosquito dispersal formulation is coupled with a classic SEIRS-type model to capture the interaction between malaria dynamics and the life cycle of *Anopheles* vectors. The model is linked to a sophisticated ecohydrologic model to incorporate climate-driven hydrologic and ecologic processes as factors that determine mosquito population and malaria transmission dynamics. A meta-population approach is incorporated to describe the movements of vectors among discrete geographic sub-domains. The It$\hat{o}$ approximation of stochastic integrals is used to derive governing stochastic differential equations (SDEs) of the model. Then, the derived SDEs are replicated across lattice grid cells in the domain to capture temporal and spatial dynamics of malaria.

The rest of this paper is organized as follows. In the next section, the mathematical formulations of the stochastic malaria model is described. Then, numerical simulations of this model using topographic and reanalysis data for a case study in Kenya are presented. Finally, the paper closes with discussion of the key points.

## Methods: model description

**Fig. 1:**
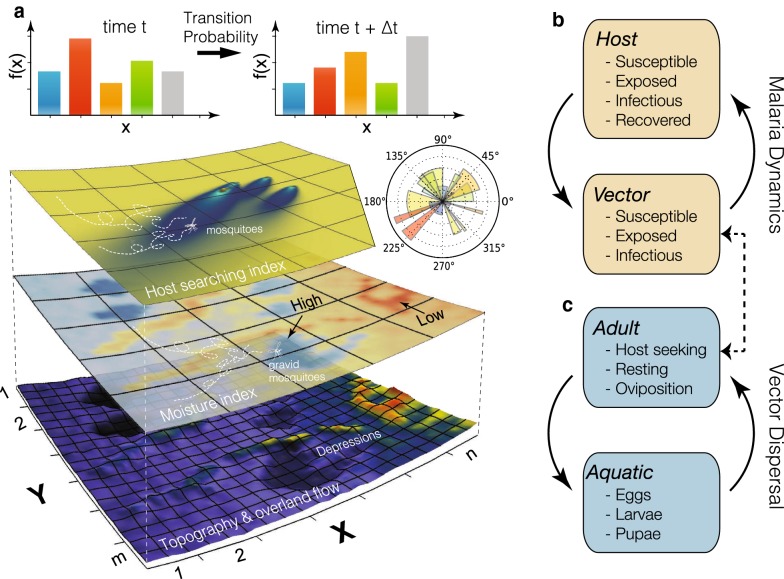
Schematic of the SLIM model that couples a vector dispersal model with a malaria dynamics formulation. **a** Ponding and moisture index obtained from an ecohydrologic model (Dhara) provide the habitat for gravid female *Anopheles* to deposit eggs. The dispersal of host-seeking mosquitoes is based on a host searching index calculated as a function of human density in each grid cell. The rates of movement of mosquitoes among adjacent grid cells are diffusive. In each cell, the changes of sub-populations (x-axes) in vector dispersal and malaria dynamics models over time period $\Delta t$ are described by transition probabilities. **b** Sub-population of compartmental malaria dynamics model in each grid cell. **c** Sub-population of vector dynamics model in each grid cell. The vector dispersal and malaria dynamics models share the adult vector population which affects both the aquatic density and malaria transmission in human hosts

SLIM consists of two stochastic time-continuous, space-discrete models: one for vector dispersal that represents the entomological life cycles of female *Anopheles*, and a second for SEIRS malaria dynamics that simulates the circulation of the pathogen between host and vector populations (see Fig. 1). The model is driven by the distribution of human population (density, locations), atmospheric forcing (air temperature, precipitation), and hydrological conditions (soil moisture distribution and ponding persistence in topographic depressions). The spread of malaria in time and space is modelled as follows. Let $\mathbb {D}$ be a bounded domain in $\mathbb {R}^2$ and let $m,n \ge 1$ be integers. $\mathbb {D}$ is partitioned into uniform and rectangular lattice grid indexed as $\{\zeta := (i,j) \in \mathbb {Z}^2_{+} : i \le n, j \le m\}$. In each grid cell $\zeta$, a vector dispersal model for mosquito population dynamics is coupled with a SEIRS formulation for malaria transmission. It is assumed that mosquitoes in aquatic stages are immobile, but adult mosquitoes can move between adjacent grid cells, spreading the pathogen.

### Vector dispersal model

The deterministic vector dispersal model of Lutambi et al. [28] that serves as the basis for constructing the stochastic formulation is described first. The life cycle of the female mosquito has six distinct phases, including three aquatic stages: egg (*E*), larval (*L*), pupal (*P*); and three adult stages: host-seeking ($A_{h}$), resting ($A_{r}$), and oviposition site searching ($A_{o}$). Each phase of this model, hereafter referred to as ELPAs, is subject to fluctuations due to recruitment, mortality, and progression of survivors into the next states. $A_{h}$ and $A_{o}$ are further affected by the movement of mosquitoes in space. For $t > t_0$ and $\forall \zeta \in \mathbb {D}$, ELPAs is given as a set of dynamical equations as described below. The summary of key parameters presented in ELPAs and their ranges of values are given in Table 1.

**Table 1:** Description and values of parameters of the ELPA$_{s}$ model. Modified from [28]

Name	Description	Unit	Range
*b*	Integer number of female eggs laid per oviposition	−	50–300
$\psi ^\mathsf {W}_{\zeta }$	50% of the eggs are assumed to hatch into female mosquitoes parameter represent water availability in cell $\zeta$	−	0.0–1.0
$\psi ^\mathsf {H}_{\zeta }$	Binary parameter represent human presence in cell $\zeta$	−	0–1
$\rho _E$	Egg hatching rate into larvae	day$^{-1}$	0.33–1.0
$\rho _L$	Rate at which larvae develop into pupae	day$^{-1}$	0.08–0.17
$\rho _P$	Rate at which pupae develop into adult/emergence rate	day$^{-1}$	0.33–1.0
$\mu _E$	Egg mortality rate	day$^{-1}$	0.32–0.80
$\mu _{L_1}$	Natural mortality rate of larvae	day$^{-1}$	0.30–0.58
$\mu _{L_2}$	Density-dependent mortality rate of larvae	day$^{-1}$mosq$^{-1}$	0.0–1.0
$\mu _P$	Pupae mortality rate	day$^{-1}$	0.22–0.52
$\rho _{A_h}$	Rate at which host-seeking mosquitoes enter the resting state	day$^{-1}$	0.322–0.598
$\rho _{A_r}$	Rate at which resting mosquitoes enter oviposition searching state	day$^{-1}$	0.30–0.56
$\rho _{A_o}$	Oviposition rate	day$^{-1}$	3.0–4.0
$\mu _{A_h}$	Mortality rate of mosquitoes of searching for hosts	day$^{-1}$	0.125–0.233
$\mu _{A_r}$	Mortality rate of resting mosquitoes	day$^{-1}$	0.0034–0.01
$\mu _{A_o}$	Mortality rate of mosquitoes searching for oviposition sites	day$^{-1}$	0.41–0.56

- The rate of change in egg population ($E_{\zeta }$) as a function of oviposition, egg mortality and hatching:
  1a $$\begin{aligned} \frac{dE_{\zeta }}{dt} = b \psi ^\mathsf {W}_{\zeta } \rho _{A_o} A_{o,\zeta } - \left( \mu _{E} + \rho _{E} \right) E_{\zeta } \end{aligned}$$
  where *b* is the average number of eggs laid during an oviposition with 1:1 sex ratio; $\rho _{A_o}$ is the rate at which eggs are oviposited by gravid mosquitoes; $\mu _{E}$ is egg mortality rate; and $\rho _{E}$ is the hatching rate into larvae. The term $\psi ^\mathsf {W}_{\zeta }$ on the right hand side represents water availability in a particular cell $\zeta$ and is discussed later.
- The rate of change in larvae population ($L_{\zeta }$) as a function of egg population, larval mortality and maturation into pupae:
  1b $$\begin{aligned} \frac{dL_{\zeta }}{dt} = \rho _{E} E_{\zeta } - \left( \mu _{L_1} + \mu _{L_2} L_{\zeta } + \rho _{L} \right) L_{\zeta } \end{aligned}$$
  where $\rho _{L}$ is the progression rate from larvae to pupae; $\mu _{L_1}$ and $\mu _{L_2}$ represent natural and density-dependent death rates of larvae, respectively.
- The rate of change in pupae population ($P_{\zeta }$) as a function of larval maturation, pupal mortality, and emergence into adults:
  1c $$\begin{aligned} \frac{dP_{\zeta }}{dt} = \rho _{L} L_{\zeta } - \left( \mu _{P} + \rho _{P} \right) P_{\zeta } \end{aligned}$$
  where $\mu _{P}$ is the mortality rate of pupae, and $\rho _{P}$ represent the rate of emergence from pupae into adults.
- The rate of change in population of host-seeking adults ($A_{h,\zeta }$) as a function of pupal emergence, oviposition, mortality, and blood feeding rates:
  1d $$\begin{aligned} \begin{array}{l} \frac{{d{A_{h,\zeta }}}}{{dt}} = {\rho _P}{P_\zeta } + \psi _\zeta ^W{\rho _{{A_o}}}{A_{o\zeta }} - \left( {{\mu _{{A_h}}} + \psi _\zeta ^H{\rho _{{A_h}}}} \right) {A_{h,\zeta }}\\ \qquad \quad+ \left( {\sum \limits _{\zeta ' \in {\mathcal {N}}} {\omega _{\zeta ':\zeta }^H} } \right) {A_{h,\zeta '}} - \left( {\sum \limits _{\zeta ' \in \mathcal{N}} {\omega _{\zeta :\zeta '}^H} \times {A_{h,\zeta }}} \right) \end{array} \end{aligned}$$
  where $\mu _{A_h}$ is the death rate of host-seeking adults; and $\rho _{A_h}$ is the rate at which they enter a resting state after blood feeding. Host-seeking adults can spread to adjacent cells for searching human host in which $\omega ^\mathsf {H} _{\zeta _1:\zeta _2}$ represents their movement rate from cell $\zeta _1$ to $\zeta _2$ modelled as a decreasing exponential function of human population and average area of cell $\zeta _1$ and $\zeta _2$ (see [28]); $\mathcal {N}$ denotes the neighbors of the cell under consideration. The last two terms in (1d) represent the movements of vectors from all neighbors $\zeta '$ into cell $\zeta$ and vice versa, respectively. After laying eggs, gravid mosquitoes return to the host-seeking state for subsequent blood feeding.
- The rate of change in population of resting adults ($A_{r,\zeta }$) as a function of blood feeding, mortality, and protein digestion rates:
  1e $$\begin{aligned} \frac{dA_{r,\zeta }}{dt} = \psi ^\mathsf {H}_{\zeta } \rho _{A_h} A_{h,\zeta } - \left( \mu _{A_r} + \rho _{A_r} \right) A_{r,\zeta } \end{aligned}$$
  where $\mu _{A_r}$ is the death rate of resting adults; and $\rho _{A_r}$ is the progression rate at which the survivors enter the oviposition site searching phase. In the resting state, female mosquitoes are usually dormant to digest protein.
- The rate of change in population of oviposition site searching adults ($A_{o,\zeta }$) as a function of emergence, oviposition, mortality, and digestion rates:
  1f $$\begin{aligned} \frac{dA_{o,\zeta }}{dt}&= \rho _{r} A_{r,\zeta } - (\mu _{A_o} + \psi ^\mathsf {W}_{\zeta } \rho _{A_o}) A_{o,\zeta } \nonumber \\&\quad + \left( \sum _{\zeta ' \in \mathcal {N}} \omega ^{\mathsf {W}}_{\zeta ':\zeta } \right) A_{o,\zeta '} - \left( \sum _{\zeta ' \in \mathcal {N}} \omega _{\zeta :\zeta '}^{\mathsf {W}} \times A_{o,\zeta } \right) \end{aligned}$$
  where $\mu _{A_o}$ is the death rate of gravid female mosquitoes. Vectors in ovipositing state also spread in space to find water for oviposition in which $\omega ^\mathsf {W} _{\zeta _1:\zeta _2}$ represents their movement rate from cell $\zeta _1$ to $\zeta _2$ modelled as a decreasing exponential function of surface soil moisture and average area of cell $\zeta _1$ and $\zeta _2$ [28].

The above model (1) is well-posed in a positively invariant domain:

$$\begin{aligned} \Omega = \{E_{\zeta }, L_{\zeta }, P_{\zeta }, A_{h,\zeta }, A_{r,\zeta }, A_{o,\zeta } \} \subseteq \mathbb {R}_{\ge 0}^{6\times m \times n}, \quad \zeta \in \mathbb {D} \end{aligned}$$

Unlike the original model [28], environmental variability is further incorporated to the developmental rates of aquatic mosquitoes using the temperature relationships [29]:

2 $$\begin{aligned} \rho _k = r_k(T_a) \Delta t \end{aligned}$$

for $k=\{ E, L, P\}$, where $T_a$ is the mean air temperature (*K*) over the time interval $\Delta t$; and $r_k(T_a) : \mathbb {R} \mapsto \mathbb {R}$ is the development rate per unit time at $T_a$. Details for the temperature dependencies of egg, larval, and pupal populations are presented in Depinay et al. [29].

Although the ELPAs model offers a simple approach to incorporate the effects of vector mobility on spatial population dynamics of vectors in heterogeneous environments, it does not account for fluctuations dominated by randomness in population events and environmental conditions. In some cases, such fluctuations may result in critical state transitions of vector population dynamics. This fact highlights the need to develop stochastic tools to address the complexities arising from vector population dynamics.

A continuous SDE model for the dynamical system (1) is developed in the following way (see Chapter 5, ref [30] for more details about the approach to stochastic modelling applied herein). First, it is assumed that there is demographic variability due to births, deaths, and transitions between the states in (1). Second, a discrete (Markov chain) stochastic model for (1) is constructed. For small interval $\Delta t$, we then identify all possible changes and their corresponding transition probabilities for the discrete stochastic processes. Third, the expected changes and the covariance matrix of changes of these processes are determined. Finally, the continuous SDE model for (1), hereafter referred to as S-ELPAs, is inferred by similarities in the forward Kolmogorov equations between the discrete and continuous stochastic processes [30, 31]. Note that solutions of the discrete Markov chain and continuous S-ELPAs models approximately have the same probability distribution.

**Fig. 2:**
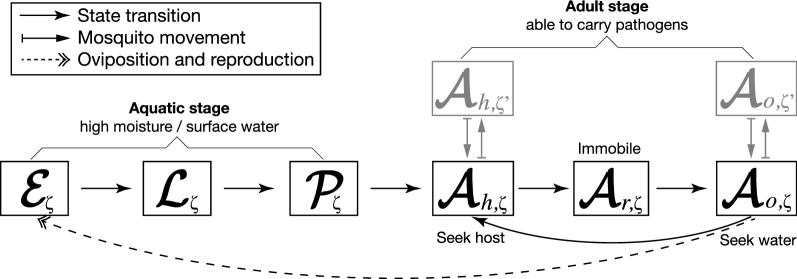
Schematic representation of *Anopheles* life and feeding cycles. The first three stages are aquatic. The last three stages are adult, which are able to carry the pathogens (Modified from Lutambi et al. [28])

Let $\mathcal {E}_{\zeta }(t)$, $\mathcal {L}_{\zeta }(t)$, $\mathcal {P}_{\zeta }(t)$, $\mathcal {A}_{h,\zeta }(t)$, $\mathcal {A}_{r,\zeta }(t)$, $\mathcal {A}_{o,\zeta }(t) \in \mathbb {R}_{\ge 0}$ denote continuous random variables for the density of eggs, larvae, pupae, host-seeking adults, resting adults, and oviposition site searching adults in a grid cell $\zeta$, respectively (see Fig. 2). The S-ELPAs model depends on the state variables:

3 $$\begin{aligned} {\varvec{X}}(t) = \big \{ \varvec{\mathcal {E}}(t), \varvec{\mathcal {L}}(t), \varvec{\mathcal {P}}(t), \varvec{\mathcal {A}}_{h}(t), \varvec{\mathcal {A}}_{r}(t), \varvec{\mathcal {A}}_{o}(t) \big \}^T \subseteq \mathbb {R}^{6\times m \times n}_{\ge 0}, \end{aligned}$$

where $\mathbf{{X}}_{k}(t) = \{X_{k,\zeta }(t) \in \mathbb {R} : \zeta \in \mathbb {D} \} \subseteq \mathbb {R}^{m \times n}_{\ge 0}$ for $k=1,\ldots ,6$ in which $\mathbf{{X}}_{k}(t)$ has an associated probability density function ${{p}}_{k}(x, t)$:

4 $$\begin{aligned} P\{a \le {\mathbf{X}}_{k}(t) \le b\} = \int _a^b {p}_{k}(x,t)dx, \end{aligned}$$

Let $\Delta {\mathbf{X}}(t) = {\mathbf{X}}(t+\Delta t) - {\mathbf{X}}(t)$. For small $\Delta t$, there are 13 possible unit changes in $\Delta X_{k,\zeta }(t)$ of the discrete stochastic model associated with different probabilities in each cell $\zeta$ (see Table 2). The It$\hat{o}$ stochastic differential equations for the S-ELPAs model are given as follow [30]:

5 $$\begin{aligned} d{\mathbf{X}}(t) = {\mathbf{F}}^x\big ( t,{\mathbf{X}}(t),{\mathbf{p}}^x(t, {\mathbf{C}^x}(t)) \big )dt + {\mathbf{G}}^x\big (t,{\mathbf{X}}(t), {\mathbf{p}}^x(t, {\mathbf{C}^x}(t)) \big ) \cdot d{\mathbf{W}}(t) \end{aligned}$$

where ${\mathbf{F}}^x : \mathbb {R}^{6\times m \times n} \rightarrow \mathbb {R}^{6\times m \times n}$; ${\mathbf{G}}^x : \mathbb {R}^{6\times 13 \times m \times n} \rightarrow \mathbb {R}^{6 \times 13 \times m \times n}$; ${\mathbf{W}}(t) \subset \mathbb {R}^{13 \times m \times n}$ is the matrix of independent Wiener processes; and ${\mathbf{p}}^x \subseteq \mathbb {R}^{m \times n}$ is the matrix of parameters that are functions of time *t* and climatic, anthropogenic, and entomological factors ${\mathbf{C}^x}(t) \subseteq \mathbb {R}^{m \times n}$.

**Table 2:** Probabilities associated with changes in ELPA$_{s}$ model

*l*	Change, $\Delta X^{l}_{k,\zeta }(t)$	Probability, $p^{l}_{k,\zeta }(t)$	Description
1	$[1, 0, 0, 0, 0, 0]^T$	$b \psi ^{\mathsf {W}}_{\zeta } \rho _{A_o} \mathcal {A}_{o,\zeta } \Delta t$	A new egg *E* is deposited by $A_o$
2	$[-1, 0, 0, 0, 0, 0]^T$	$\mu _E \mathcal {E}_{\zeta } \Delta t$	An egg *E* dies
3	$[-1, 1, 0, 0, 0, 0]^T$	$\rho _E \mathcal {E}_{\zeta } \Delta t$	An egg *E* hatches into a larva *L*
4	$[0, -1, 0, 0, 0, 0]^T$	$(\mu _{L_1} + \mu _{L_2} \mathcal {L}_{\zeta } ) \mathcal {L}_{\zeta } \Delta t$	A larva *L* dies
5	$[0, -1, 1, 0, 0, 0]^T$	$\rho _L \mathcal {L}_{\zeta } \Delta t$	A larva *L* develops into a pupa *P*
6	$[0, 0, -1, 0, 0, 0]^T$	$\mu _P \mathcal {P}_{\zeta } \Delta t$	A pupa *P* dies
7	$[0, 0, -1, 1, 0, 0]^T$	$\rho _P \mathcal {P}_{\zeta } \Delta t$	A pupa *P* develops into a host-seeking adult $A_h$
8	$[0, 0, 0, 1, 0, -1]^T$	$\psi ^\mathsf {W}_{\zeta } \rho _{A_o} \mathcal {A}_{o,\zeta } \Delta t$	An oviposition adult $A_o$ enters host-seeking state
9	$[0, 0, 0, -1, 0, 0]^T$	$\mu _{A_h} \mathcal {A}_{h,\zeta } \Delta t$	A host-seeking adult $A_h$ dies
10	$[0, 0, 0, -1, 1, 0]^T$	$\psi ^\mathsf {H}_{\zeta } \rho _{A_h} \mathcal {A}_{h,\zeta } \Delta t$	A host-seeking adult $A_h$ enters resting state
11	$[0, 0, 0, 0, -1, 0]^T$	$\mu _{A_r} \mathcal {A}_{r,\zeta } \Delta t$	A resting adult $A_r$ dies
12	$[0, 0, 0, 0, -1, 1]^T$	$\rho _{A_r} \mathcal {A}_{r,\zeta } \Delta t$	A resting adult $A_r$ enters oviposition searching state
13	$[0, 0, 0, 0, 0, -1]^T$	$\mu _{A_o} \mathcal {A}_{o,\zeta } \Delta t$	An oviposition searching adult $A_o$ dies

The drift term ${\mathbf{F}}^x$ in (5), to order $\Delta t$, is the expectation of all possible changes in the discrete stochastic model computed as:

6 $$\begin{aligned} \varvec{F}^x=\, & {} \langle \Delta \varvec{X}(t) \rangle \equiv \sum _{l=1}^{13} \varvec{p}_{k,l,\zeta }(t) \Delta \varvec{X}_{k,l,\zeta }(t) \nonumber \\= & {} \begin{pmatrix} b \varvec{\psi }^\mathsf {W} \rho _{A_o} \varvec{\mathcal {A}}_{o} - (\mu _E + \rho _E) \varvec{\mathcal {E}} \\ \rho _{E} \varvec{\mathcal {E}} - (\mu _{L_1} + \mu _{L_2} \varvec{\mathcal {L}} + \rho _{L}) \varvec{\mathcal {L}} \\ \rho _{L} \varvec{\mathcal {L}} - (\mu _{P} + \rho _{P}) \varvec{\mathcal {P}} \\ \rho _{P} \varvec{\mathcal {P}} + \varvec{\psi }^\mathsf {W} \rho _{A_o} \varvec{\mathcal {A}}_{o} - (\mu _{A_h} + \varvec{\psi }^\mathsf {H} \rho _{A_h}) \varvec{\mathcal {A}}_{h} \\ \varvec{\psi }^\mathsf {H} \rho _{A_h} \varvec{\mathcal {A}}_{h} - (\mu _{A_r} + \rho _{A_r}) \varvec{\mathcal {A}}_{r} \\ \rho _{r} \varvec{\mathcal {A}}_{r,} - (\mu _{A_o} + \varvec{\psi }^\mathsf {W} \rho _{A_o}) \varvec{\mathcal {A}}_{o} \end{pmatrix} \Delta t \end{aligned}$$

for $$k = 1,\ldots,6$$ and $$\forall \zeta \in \mathbb {D}$$.

Additional Wiener processes are included into the stochastic systems to simplify the derivation of the diffusion term ${{\varvec{G}}}^x$ [30, 31], written as follows:

7 $$\begin{aligned} \varvec{G}^x = \{ G^x_{k,l,\zeta }\ : \zeta \in \mathbb {D}, 1 \le k \le 6, 1 \le l \le 13 \} \end{aligned}$$

in which the covariance matrix associated with the transition probabilities to form the diffusion term is computed as:

8 $$\begin{aligned} G^{x}_{k,l,\zeta } =\Delta X_{k,l,\zeta }(t) \sqrt{p_{k,l,\zeta }(t)}, \quad \forall \zeta \in \mathbb {D} \end{aligned}$$

The diffusion term ${{\varvec{G}}}^x_{\zeta }$ in the S-ELPAs model is obtained as:

9 $$\begin{aligned} G^{x,T}_{\zeta } &= \begin{pmatrix} \sqrt{b \psi ^{\mathsf {W}}_{\zeta } \rho _{A_o} \mathcal {A}_{o,\zeta } \Delta t} \\ \sqrt{\mu _E \mathcal {E}_{\zeta } \Delta t} \\ \sqrt{\rho _E \mathcal {E}_{\zeta } \Delta t} \\ \sqrt{(\mu _{L_1} + \mu _{L_2} \mathcal {L}_{\zeta } ) \mathcal {L}_{\zeta } \Delta t} \\ \sqrt{\rho _L \mathcal {L}_{\zeta } \Delta t} \\ \sqrt{\mu _P \mathcal {P}_{\zeta } \Delta t} \\ \sqrt{\rho _P \mathcal {P}_{\zeta } \Delta t} \\ \sqrt{\psi ^\mathsf {W}_{\zeta } \rho _{A_o} \mathcal {A}_{o,\zeta } \Delta t} \\ \sqrt{\mu _{A_h} \mathcal {A}_{h,\zeta } \Delta t} \\ \sqrt{\psi ^\mathsf {H}_{\zeta } \rho _{A_h} \mathcal {A}_{h,\zeta } \Delta t} \\ \sqrt{\mu _{A_r} \mathcal {A}_{r,\zeta } \Delta t} \\ \sqrt{\rho _{A_r} \mathcal {A}_{r,\zeta } \Delta t} \\ \sqrt{\mu _{A_o} \mathcal {A}_{o,\zeta } \Delta t} \end{pmatrix} \times \varvec{ I}_{13} \times \begin{pmatrix} 1 &{} 0 &{} 0 &{} 0 &{} 0 &{} 0 \\ -1 &{} 0 &{} 0 &{} 0 &{} 0 &{} 0 \\ -1 &{} 1 &{} 0 &{} 0 &{} 0 &{} 0 \\ 0 &{} -1 &{} 0 &{} 0 &{} 0 &{} 0 \\ 0 &{} -1 &{} 1 &{} 0 &{} 0 &{} 0 \\ 0 &{} 0 &{} -1 &{} 0 &{} 0 &{} 0 \\ 0 &{} 0 &{} -1 &{} 1 &{} 0 &{} 0 \\ 0 &{} 0 &{} 0 &{} 1 &{} 0 &{}-1 \\ 0 &{} 0 &{} 0 &{} -1 &{} 0 &{} 0 \\ 0 &{} 0 &{} 0 &{} -1 &{} 1 &{} 0 \\ 0 &{} 0 &{} 0 &{} 0 &{} -1 &{} 0 \\ 0 &{} 0 &{} 0 &{} 0 &{} -1 &{} 1 \\ 0 &{} 0 &{} 0 &{} 0 &{} 0 &{}-1 \end{pmatrix} \\ &= \begin{pmatrix} \sqrt{b \varvec{\psi }^\mathsf {W} \rho _{A_{o}} \mathcal {A}_{o,\zeta }} &{} 0 &{} 0 &{} 0 &{} 0 &{} 0 \\ -\sqrt{\mu _E \mathcal {E_{\zeta }}} &{} 0 &{} 0 &{} 0 &{} 0 &{} 0 \\ -\sqrt{\rho _E \mathcal {E_{\zeta }}} &{} \sqrt{\rho _E \mathcal {E_{\zeta }}} &{} 0 &{} 0 &{} 0 &{} 0 \\ 0 &{} -\sqrt{\mu _{L_1}\mathcal {L}_{\zeta }\!+\!\mu _{L_2}\mathcal {L}^2_{\zeta }} &{} 0 &{} 0 &{} 0 &{} 0 \\ 0 &{} -\sqrt{\rho _L \mathcal {L_{\zeta }}} &{} \sqrt{\rho _L \mathcal {L_{\zeta }}} &{} 0 &{} 0 &{} 0 \\ 0 &{} 0 &{} -\sqrt{\mu _P \mathcal {P_{\zeta }}} &{} 0 &{} 0 &{} 0 \\ 0 &{} 0 &{} -\sqrt{\rho _P \mathcal {P_{\zeta }}} &{} \sqrt{\rho _P \mathcal {P_{\zeta }}} &{} 0 &{} 0 \\ 0 &{} 0 &{} 0 &{} \sqrt{\psi ^\mathsf {W} \rho _{A_o} \mathcal {A}_{o,\zeta }} &{} 0 &{} -\sqrt{\psi ^\mathsf {W} \rho _{A_o} \mathcal {A}_{o,\zeta }} \\ 0 &{} 0 &{} 0 &{} -\sqrt{\mu _{A_h} \mathcal {A}_{h,\zeta }} &{} 0 &{} 0 \\ 0 &{} 0 &{} 0 &{} -\sqrt{\psi ^\mathsf {H} \rho _{A_h} \mathcal {A}_{h,\zeta }} &{} \sqrt{\psi ^\mathsf {H} \rho _{A_h} \mathcal {A}_{h,\zeta }} &{} 0 \\ 0 &{} 0 &{} 0 &{} 0 &{} -\sqrt{\mu _{A_r} \mathcal {A}_{r,\zeta }} &{} 0 \\ 0 &{} 0 &{} 0 &{} 0 &{} -\sqrt{\rho _{A_r} \mathcal {A}_{r,\zeta }} &{} \sqrt{\rho _{A_r} \mathcal {A}_{r,\zeta }} \\ 0 &{} 0 &{} 0 &{} 0 &{} 0 &{} -\sqrt{\mu _{A_o} \mathcal {A}_{o,\zeta }} \end{pmatrix} \sqrt{\Delta t} \end{aligned}$$

where ${{\varvec{I}}}_{13}$ is the 13 $\times 13$ identity.

The S-ELPAs model provides the basis to capture spatial variation of mosquito population dynamics. It incorporates random processes and heterogeneity in both densities of human hosts and breeding sites for the feeding and life cycles of the vectors. S-ELPAs is coupled with a stochastic SEIRS formulation presented below for malaria transmission dynamics.

### Malaria transmission model

The S-ELPAs model described above extends the deterministic ELPAs model to incorporate stochastic variability associated with population and spatial dynamics of the mosquitoes. Similarly, a stochastic version of SEIRS formulations is developed to capture the variability associated with the circulations of malaria parasites between human and vector populations. The aim is then to combine and link them to an ecohydrological model that explicitly considers climate-driven hydrologic factors for simulating mosquito population dynamics and malaria transmission.

The lumped, deterministic SEIRS formulations [9, 10] shown below is extended to develop a stochastic malaria model. The human host population is divided into four distinct classes: susceptible ($S_h$), exposed ($E_h$), infectious ($I_h$), and recovered ($R_h$). The adult vector population is divided into three classes: susceptible ($S_v$), exposed ($E_v$), and infectious ($I_v$). Here, the aquatic stages of vectors are not considered in SEIRS models. For $t > t_0$ and $\forall \zeta \in \mathbb {D}$, the deterministic SEIRS model is given by another set of ODEs that characterize:

- The rate of change in susceptible host ($S_{h,\zeta }$) as a function of immigration, birth, human infection, recovery from infection, and human mortality:
  10a $$\begin{aligned} \frac{dS_{h,\zeta }}{dt} =\,& \Lambda _h + \psi _h N_{h,\zeta } + \rho _h R_{h,\zeta }\\& - \lambda _{h,\zeta }(t) S_{h,\zeta } - f_h(N_{h,\zeta }) S_{h,\zeta } \end{aligned}$$
  where $\Lambda _h$ is immigration rate; and $\psi _h$ is per capita birth rate of humans; $\rho _h$ is per capita rate of losing acquired temporary immunity. Acquired temporary immunity represents the enhancement of the defense mechanism of the human host as a result of a previous encounter with the pathogen [32]. $N_{h,\zeta } = S_{h,\zeta } + E_{h,\zeta } + I_{h,\zeta } + R_{h,\zeta }$ is total population size for humans in each cell $\zeta$; $f_h(N_{h,\zeta }) = \mu _{1h} + \mu _{2h} N_{h,\zeta }$ is the human per capita death rate; and $\lambda _{h,\zeta }$ is the infection rate from mosquitoes to humans defined as:
  $$\begin{aligned} \lambda _{h,\zeta } = \frac{\sigma _v \sigma _h}{\sigma _v N_{v,\zeta } + \sigma _h N_{h,\zeta }} \times \beta _{hv} I_{v,\zeta } \end{aligned}$$
  in which $N_{v,\zeta } = S_{v,\zeta } + E_{v,\zeta } + I_{v,\zeta }$ is total population of mosquitoes in cell $\zeta$; $\sigma _v$ represents the number of times one mosquito attempt to bite humans per unit time; $\sigma _h$ is the maximum number of mosquito bites a human can have per unit time; and $\beta _{hv}$ is the probability of infection transmission from an infectious mosquito to a susceptible human, given that a contact between the two occurs.
- The rate of change in exposed host ($E_{h,\zeta }$) as a function of new host infections, latent period, and human mortality:
  10b $$\begin{aligned} \frac{dE_{h,\zeta }}{dt} = \lambda _{h,\zeta }(t) S_{h,\zeta } - \nu _h E_{h,\zeta } - f_h(N_{h,\zeta }) E_{h,\zeta } \end{aligned}$$
  where $\nu _h$ is per capita rate of progression of humans from exposed to infectious state.
- The rate of change in infected host ($I_{h,\zeta }$) as a function of latent period, recovery and human mortality rates:
  10c $$\begin{aligned} \frac{dI_{h,\zeta }}{dt} = \nu _h E_{h,\zeta } - \gamma _h I_{h,\zeta } - f_h(N_{h,\zeta }) I_{h,\zeta } - \delta _h I_{h,\zeta } \end{aligned}$$
  where $\gamma _h$ represents per capita recovery rate for humans from infectious to recovered states; and $\delta _h$ represents per capita disease-induced death rate for humans.
- The rate of change in recovered host ($R_{h,\zeta }$) as a function of recovery, immunity loss, and human mortality:
  10d $$\begin{aligned} \frac{dR_{h,\zeta }}{dt} = \gamma _h I_{h,\zeta } - \rho _h R_{h,\zeta } - f_h(N_{h,\zeta }) R_{h,\zeta } \end{aligned}$$
- The rate of change in susceptible vector ($S_{v,\zeta }$) as a function of reproduction, vector infection, and vector mortality:
  10e $$\begin{aligned} \frac{dS_{v,\zeta }}{dt} = \psi _v N_{v,\zeta } - \lambda _{v,\zeta }(t) S_{v,\zeta } - f_v(N_{v,\zeta }) S_{v,\zeta } \end{aligned}$$
  where $\psi _v$ represents per capita birth rate of the vectors; $f_v(N_{v,\zeta }) = \mu _{1v} + \mu _{2v} N_{v,\zeta }$ is the per capita death rate for vectors in each cell $\zeta$; and $\lambda _{v,\zeta }$ is the infection rate from humans to mosquitoes defined as:
  $$\begin{aligned} \lambda _{v,\zeta } = \frac{\sigma _v \sigma _h}{\sigma _v N_{v,\zeta } + \sigma _h N_{h,\zeta }} \times \left( \beta _{vh} I_{h,\zeta } + \tilde{\beta _{vh}} R_{h,\zeta } \right) \end{aligned}$$
  where $\beta _{vh}$ and $\tilde{\beta }_{hv}$ represent the transmission probability of infection from an infectious and a recovered human, respectively, to a susceptible mosquito, given that a contact between them occurs.
- The rate of change in exposed vector ($E_{v,\zeta }$) as a function of new vector infection, vector latent period, and vector mortality:
  10f $$\begin{aligned} \frac{dE_{v,\zeta }}{dt} = \lambda _{v,\zeta }(t) S_{v,\zeta } - \nu _v E_{v,\zeta } - f_v(N_{v,\zeta }) E_{v,\zeta } \end{aligned}$$
  where $\nu _v$ is per capita rate of progression of mosquitoes from the exposed state to the infectious state.
- The rate of change in infected vector ($I_{v,\zeta }$) as a function of latent period and mortality:
  10g $$\begin{aligned} \frac{dI_{v,\zeta }}{dt} = \nu _v E_{v,\zeta } - f_v(N_{v,\zeta }) I_{v,\zeta } \end{aligned}$$

**Table 3:** The parameters for SEIRS malaria model. From [10]

Name	Description	Unit^a^
$\Lambda _h$	Immigration rate of humans	H $\times$ T$^{-1}$
$\psi _h$	Per capita birth rate of humans	T$^{-1}$
$\psi _v$	Per capita birth rate of mosquitoes	T$^{-1}$
$\sigma _v$	Number of times one mosquito would want to bite humans per unit time, if humans were freely available. This is a function of the mosquito’s gonotrophic cycle (the amount of time a mosquito requires to produce eggs) and its anthropophilic rate (its preference for human blood)	T$^{-1}$
$\sigma _h$	The maximum number of mosquito bites a human can have per unit time. This is a function of the human’s exposed surface area	T$^{-1}$
$\beta _{hv}$	Probability of transmission of infection from an infectious mosquito to a susceptible human, given that a contact between the two occurs	−
$\beta _{vh}$	Probability of transmission of infection from an infectious human to a susceptible mosquito, given that a contact between the two occurs	−
$\tilde{\beta }_{hv}$	Probability of transmission of infection from a recovered (asymptomatic carrier) human to a susceptible mosquito, given that a contact between the two occurs	−
$\nu _h$	Per capita rate of progression of humans from the exposed state to the infectious state. $1/\nu _h$ is the average duration of the latent period	T$^{-1}$
$\nu _v$	Per capita rate of progression of mosquitoes from the exposed state to the infectious state. $1/\nu _v$ is the average duration of the latent period	T$^{-1}$
$\gamma _h$	Per capita recovery rate for humans from the infectious state to the recovered state. $1/\gamma _h$ is the average duration of the infectious period	T$^{-1}$
$\delta _h$	Per capita disease-induced death rate for humans	T$^{-1}$
$\rho _h$	Per capita rate of loss of acquired temporary immunity for humans. $1/\rho _h$ is the average duration of the immune period	T$^{-1}$
$\mu _{1h}$	Density-independent part of the death (and emigration) rate for humans	T$^{-1}$
$\mu _{2h}$	Density-dependent part of the death (and emigration) rate for humans	H $\times$ T$^{-1}$
$\mu _{1h}$	Density-independent part of the death (and emigration) rate for mosquitoes	T$^{-1}$
$\mu _{2h}$	Density-dependent part of the death (and emigration) rate for mosquitoes	M $\times$ T$^{-1}$

^a^In the Unit, H represents humans, M represents mosquitoes, and T represents time

A summary of parameters associated with (10) are shown in Table 3. All parameters described are strictly positive with the exception of the disease-induced death rate, $\delta _h$, which is non-negative [10]. The model is well-posed in a positively invariant domain:

$$\begin{aligned} \Omega = \{S_{h,\zeta }, E_{h,\zeta }, I_{h,\zeta }, R_{h,\zeta }, S_{v,\zeta }, E_{v,\zeta }, I_{v,\zeta } \} \subseteq \mathbb {R}_{\ge 0}^{7\times m \times n}, \quad \zeta \in \mathbb {D} \end{aligned}$$

The development rate of *Plasmodium* parasites within humans $\nu _h$, or the intrinsic incubation period, is assumed to be temperature independent.

However, the development rate of *Plasmodium* within mosquitoes $\nu _v$, or extrinsic incubation period, is highly dependent on air temperature. Unlike the original models [9, 10], the fitted temperature-development function for the development rate of parasites within the vector [33–35] is also incorporated to the extended model:

11 $$\begin{aligned} \nu _v(T_a) = 0.000112 \times T_a \times (T_a-15.384) \sqrt{35 - T_a} \end{aligned}$$

where $\nu _v(T_a) : \mathbb {R} \mapsto \mathbb {R}$ is the progression rate of mosquitoes from exposed to infectious state and $T_a \le 35$.

Next, the SDE model from the ODE systems described above is derived to incorporate random fluctuations of malaria transmission. It is again assumed that there is variability described by random noise in the transitions between states in (10). Similarly, a discrete (Markov chain) stochastic model is first developed and the expected changes and covariance matrix of changes of the discrete stochastic processes are identified. Then, the continuous stochastic SEIRS model, hereafter referred to as S-SEIRS, is derived. Solutions of the discrete Markov chain and continuous S-SEIRS models approximately have the same probability distribution as well.

**Fig. 3:**
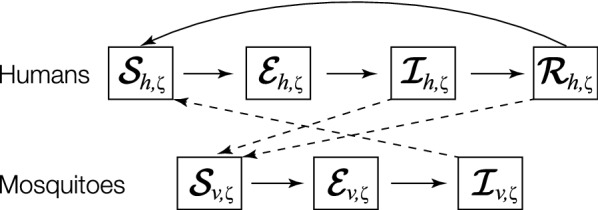
Schematic representation of malaria transmission. The model divides the human population into four classes: susceptible, $S_h$; exposed, $E_h$; infectious, $I_h$; and recovered (immune), $R_h$. Vector population is divided into three classes: susceptible, $S_v$; exposed, $E_v$; and infectious, $I_v$. Both species follow a logistic population model, with humans having additional immigration and disease-induced death. Birth, death, and migration into and out of the population are not shown in the figure (Adapted from Chitnis et al. [10])

Let $\mathcal {S}_{i,\zeta }(t)$, $\mathcal {E}_{i,\zeta }(t)$, $\mathcal {I}_{i,\zeta }(t)$, and $\mathcal {R}_{h,\zeta }(t)$ denote continuous random variables for the density of susceptible, exposed, infectious for human ($i=h$) and vector ($i=v$), and recovered human, respectively (Fig. 3). The S-SEIRS model depends on the state variables:

12 $$\begin{aligned} \varvec{Y}(t) = \left\{ \varvec{\mathcal {S}}_h(t), \varvec{\mathcal {E}}_h(t), \varvec{\mathcal {I}}_h(t), \varvec{\mathcal {R}}_h(t), \varvec{\mathcal {S}}_v(t), \varvec{\mathcal {E}}_v(t), \varvec{\mathcal {I}}_v(t) \right\} \subseteq \mathbb {R}^{7\times m \times n}_{\ge 0}, \end{aligned}$$

where ${{\varvec{Y}}}_{k}(t) = \{Y_{k,\zeta }(t) \in \mathbb {R} : \zeta \in \mathbb {D} \} \subseteq \mathbb {R}^{m \times n}_{\ge 0}$ for $k=1,\ldots ,7$ in which ${{\varvec{Y}}}_{k}(t)$ has an associated probability density function ${{\varvec{p}}}_{k}(y, t)$:

13 $$\begin{aligned} P\{a \le {{\varvec{Y}}}_{k}(t) \le b\} = \int _a^b {{\varvec{p}}}_{k}(y,t)dy, \end{aligned}$$

**Table 4:** Probabilities associated with changes in SEIRS model

*l*	Change, $\Delta Y^{l}_{k,\zeta }(t)$	Probability, $p^{l}_{k,\zeta }(t)$	Description
1	$[1, 0, 0, 0, 0, 0, 0]^T$	$(\Lambda _h + \psi _h N_{h,\zeta }) \Delta t$	A new host enters the human susceptible class
2	$[1, 0, 0, -1, 0, 0, 0]^T$	$\rho _h R_{h,\zeta } \Delta t$	A recovered host becomes susceptible again
3	$[-1, 1, 0, 0, 0, 0, 0]^T$	$\frac{\sigma _v \sigma _h \beta _{hv}I_{v,\zeta }S_{h,\zeta }}{\sigma _v N_{v,\zeta } + \sigma _h N_{h,\zeta }}\Delta t$	A susceptible host enters exposed state
4	$[-1, 0, 0, 0, 0, 0, 0]^T$	$(\mu _{1h} + \mu _{2h} N_{h,\zeta })S_{h,\zeta } \Delta t$	A susceptible host dies
5	$[0, -1, 1, 0, 0, 0, 0]^T$	$\nu _h E_{h,\zeta } \Delta t$	An exposed host enters infectious state
6	$[0, -1, 0, 0, 0, 0, 0]^T$	$(\mu _{1h} + \mu _{2h} N_{h,\zeta })E_{h,\zeta } \Delta t$	An exposed host dies
7	$[0, 0, -1, 1, 0, 0, 0]^T$	$\gamma _h I_{h,\zeta } \Delta t$	An infectious host enters recovered state
8	$[0, 0, -1, 0, 0, 0, 0]^T$	$(\mu _{1h} + \mu _{2h} N_{h,\zeta } + \delta _h) I_{h,\zeta } \Delta t$	An infectious host dies
9	$[0, 0, 0, -1, 0, 0, 0]^T$	$(\mu _{1h} + \mu _{2h} N_{h,\zeta }) R_{h,\zeta } \Delta t$	A recovered host dies
10	$[0, 0, 0, 0, 1, 0, 0]^T$	$\psi _v N_{v,\zeta } \Delta t$	A new mosquito enters the vector susceptible class
11	$[0, 0, 0, 0, -1, 1, 0]^T$	$\frac{\sigma _v \sigma _h \beta _{hv}I_{v,\zeta } S_{h,\zeta }}{\beta _{vh} I_{h,\zeta } + \tilde{\beta }_{vh} R_{h,\zeta }} \Delta t$	A susceptible vector enters exposed state
12	$[0, 0, 0, 0, -1, 0, 0]^T$	$(\mu _{1v} + \mu _{2v} N_{v,\zeta }) S_{v,\zeta } \Delta t$	A susceptible vector dies
13	$[0, 0, 0, 0, 0, -1, 1]^T$	$\nu _v E_{v,\zeta } \Delta t$	An exposed vector enters infectious state
14	$[0, 0, 0, 0, 0, -1, 0]^T$	$(\mu _{1v} + \mu _{2v} N_{v,\zeta }) E_{v,\zeta } \Delta t$	An exposed vector dies
15	$[0, 0, 0, 0, 0, 0, -1]^T$	$(\mu _{1v} + \mu _{2v} N_{v,\zeta }) I_{v,\zeta } \Delta t$	An infectious vector dies

Let $\Delta {\varvec{Y}}(t) = {{\varvec{Y}}}(t+\Delta t) - {{\varvec{Y}}}(t)$. For small $\Delta t$, there are 15 possible unit changes in $\Delta Y_{k,\zeta }(t)$ of the discrete stochastic processes associated with different probabilities in each grid cell $\zeta$ (Table 4). The It$\hat{o}$ stochastic differential equations for the S-SEIRS model are given as:

14 $$\begin{aligned} d{{\varvec{Y}}}(t) =\; {{\varvec{F}}}^y\big ( t,{{\varvec{Y}}}(t),{{\varvec{p}}}^y(t, {{\varvec{C}}}^y(t)) \big )dt + {{\varvec{G}}}^y\big (t,{{\varvec{Y}}}(t), {{\varvec{p}}}^y(t, {\varvec{C}}^y(t)) \big )d{{\varvec{W}}}(t) \end{aligned}$$

in which ${{\varvec{F}}}^y : \mathbb {R}^{7\times m \times n} \rightarrow \mathbb {R}^{7\times m \times n}$, ${\varvec{G}}^y : \mathbb {R}^{7\times 15 \times m \times n} \rightarrow \mathbb {R}^{7 \times 15 \times m \times n}$, ${{\varvec{W}}}(t) \subset \mathbb {R}^{15 \times m \times n}$ is matrix of independent Wiener processes, and ${{\varvec{p}}}^y \subseteq \mathbb {R}^{m \times n}$ is the matrix of parameters which are functions of time *t* and climatic and socio-economic factors ${{\varvec{C}}^y}(t) \subseteq \mathbb {R}^{m \times n}$. The drift term ${{\varvec{F}}}^y$ is calculated as:

15 $$\begin{aligned} {{\varvec{F}}}^y= & {} \langle \Delta {{\varvec{Y}}}(t) \rangle \equiv \sum _{l=1}^{15} {{\varvec{p}}}_{k,l,\zeta }(t) \times \Delta {{\varvec{Y}}}_{k,l,\zeta }(t) \nonumber \\= & {} \begin{pmatrix} {\Lambda} _h + \psi _h {\varvec{\mathcal {N}}}_h + \rho _h {\varvec{\mathcal {R}}}_h - ({\varvec{\mathcal {\lambda }}}_{h} + \mu _{1h} + \mu _{2h} {\varvec{\mathcal {N}}}_h){\varvec{\mathcal {S}}}_h \\ {\varvec{\mathcal {\lambda }}}_{h} {\varvec{\mathcal {S}}}_h - (\nu _h + \mu _{1h} + \mu _{2h} {\varvec{\mathcal {N}}}_h) {\varvec{\mathcal {E}}}_h \\ \nu _h {\varvec{\mathcal {E}}}_h - (\gamma _h + \mu _{1h} + \mu _{2h} {\varvec{\mathcal {N}}}_h + \delta _h) {\varvec{\mathcal {I}}}_h \\ \gamma _h {\varvec{\mathcal {I}}}_h - (\rho _h +\mu _{1h} + \mu _{2h} {\varvec{\mathcal {N}}}_h) {\varvec{\mathcal {R}}}_h \\ \psi _v {\varvec{\mathcal {N}}}_v - ( {\varvec{\mathcal {\lambda }}}_{v} + \mu _{1v} + \mu _{2v} {\varvec{\mathcal {N}}}_v) {\varvec{\mathcal {S}}}_v \\ {\varvec{\mathcal {\lambda }}}_{v} {\varvec{\mathcal {S}}}_v - (\nu _v + \mu _{1v} + \mu _{2v} {\varvec{\mathcal {N}}}_v) {\varvec{\mathcal {E}}}_v \\ \nu _v {\varvec{\mathcal {E}}}_v - (\mu _{1v} + \mu _{2v} {\varvec{\mathcal {N}}}_v) {\varvec{\mathcal {I}}}_v \end{pmatrix} \Delta t \end{aligned}$$

for $k = 1,\ldots,7$ and $\zeta \in \mathbb {D}$. Using a similar approach for derivation of the diffusion term in S-ELPAs model, the form of diffusion term ${{\varvec{G}}}^y$ in S-SEIRS is written as:

16 $$\begin{aligned} {{\varvec{G}}}^y = \{ G^{y}_{k,l,\zeta }\ : \zeta \in \mathbb {D}, 1 \le k \le 7, 1 \le l \le 15 \} \end{aligned}$$

in which:

17 $$\begin{aligned} G^{y}_{k,l,\zeta } =\Delta Y_{k,l,\zeta }(t) \sqrt{p_{k,l,\zeta }(t)} \end{aligned}$$

Thus, the diffusion term in the S-SEIRS model is obtained as follows:

18 $$\begin{aligned} G^{y,T}_{\zeta } = \begin{pmatrix} \sqrt{(\Lambda _h + \psi _h N_{h,\zeta }) \Delta t} \\ \sqrt{\rho _h R_{h,\zeta } \Delta t} \\ \sqrt{\frac{\sigma _v \sigma _h \beta _{hv}I_{v,\zeta }S_{h,\zeta }}{\sigma _v N_{v,\zeta } + \sigma _h N_{h,\zeta }}\Delta t} \\ \sqrt{(\mu _{1h} + \mu _{2h} N_{h,\zeta })S_{h,\zeta } \Delta t} \\ \sqrt{\nu _h E_{h,\zeta } \Delta t} \\ \sqrt{(\mu _{1h} + \mu _{2h} N_{h,\zeta })E_{h,\zeta } \Delta t} \\ \sqrt{\gamma _h I_{h,\zeta } \Delta t} \\ \sqrt{(\mu _{1h} + \mu _{2h} N_{h,\zeta } + \delta _h) I_{h,\zeta } \Delta t} \\ \sqrt{(\mu _{1h} + \mu _{2h} N_{h,\zeta }) R_{h,\zeta } \Delta t} \\ \sqrt{ \psi _v N_{v,\zeta } \Delta t} \\ \sqrt{\frac{\sigma _v \sigma _h \beta _{hv}I_{v,\zeta } S_{h,\zeta }}{\beta _{vh} I_{h,\zeta } + \tilde{\beta }_{vh} R_{h,\zeta }} \Delta t} \\ \sqrt{(\mu _{1v} + \mu _{2v} N_{v,\zeta }) S_{v,\zeta } \Delta t} \\ \sqrt{\nu _v E_{v,\zeta } \Delta t} \\ \sqrt{(\mu _{1v} + \mu _{2v} N_{v,\zeta }) E_{v,\zeta } \Delta t} \\ \sqrt{(\mu _{1v} + \mu _{2v} N_{v,\zeta }) I_{v,\zeta } \Delta t} \end{pmatrix} \times {{\varvec{I}}}_{15} \times \begin{pmatrix} 1 &{} 0 &{} 0 &{} 0 &{} 0 &{} 0 &{} 0 \\ 1 &{} 0 &{} 0 &{} -1 &{} 0 &{} 0 &{} 0 \\ -1 &{} 1 &{} 0 &{} 0 &{} 0 &{} 0 &{} 0 \\ -1 &{} 0 &{} 0 &{} 0 &{} 0 &{} 0 &{} 0 \\ 0 &{} -1 &{} 1 &{} 0 &{} 0 &{} 0 &{} 0 \\ 0 &{} -1 &{} 0 &{} 0 &{} 0 &{} 0 &{} 0 \\ 0 &{} 0 &{} -1 &{} 1 &{} 0 &{} 0 &{} 0 \\ 0 &{} 0 &{} -1 &{} 0 &{} 0 &{} 0 &{} 0 \\ 0 &{} 0 &{} 0 &{} -1 &{} 0 &{} 0 &{} 0 \\ 0 &{} 0 &{} 0 &{} 0 &{} 1 &{} 0 &{} 0 \\ 0 &{} 0 &{} 0 &{} 0 &{} -1 &{} 1 &{} 0 \\ 0 &{} 0 &{} 0 &{} 0 &{} -1 &{} 0 &{} 0 \\ 0 &{} 0 &{} 0 &{} 0 &{} 0 &{} -1 &{} 1 \\ 0 &{} 0 &{} 0 &{} 0 &{} 0 &{} -1 &{} 0 \\ 0 &{} 0 &{} 0 &{} 0 &{} 0 &{} 0 &{} -1 \end{pmatrix} = \begin{pmatrix} \sqrt{\Lambda _h\!+\!\psi _h \mathcal {N}_{h,\zeta }} &{} 0 &{} 0 &{} 0 &{} 0 &{} 0 &{}0 \\ \sqrt{\rho _h \mathcal {R}_{h,\zeta }} &{} 0 &{} 0 &{} -\sqrt{\rho _h \mathcal {R}_{h,\zeta }} &{} 0 &{} 0 &{}0 \\ -\sqrt{\lambda _{h,\zeta } \mathcal {S}_{h,\zeta }} &{} \sqrt{\lambda _{h,\zeta } \mathcal {S}_{h,\zeta }} &{} 0 &{} 0 &{} 0 &{} 0 &{}0 \\ -\sqrt{f_h \mathcal {N}_{h,\zeta } \mathcal {S}_{h,\zeta }} &{} 0 &{} 0 &{} 0 &{} 0 &{} 0 &{}0 \\ 0 &{} -\sqrt{\nu _h \mathcal {E}_{h,\zeta }} &{} \sqrt{\nu _h \mathcal {E}_{h,\zeta }} &{} 0 &{} 0 &{} 0 &{}0 \\ 0 &{} -\sqrt{f_h \mathcal {E}_{h,\zeta }} &{} 0 &{} 0 &{} 0 &{} 0 &{} 0 \\ 0 &{} 0 &{} -\sqrt{\gamma _h \mathcal {I}_{h,\zeta }} &{} \sqrt{\gamma _h \mathcal {I}_{h,\zeta }} &{} 0 &{} 0 &{} 0 \\ 0 &{} 0 &{} -\sqrt{(f_h\!+\!\delta _h) \mathcal {I}_{h,\zeta }} &{} 0 &{} 0 &{} 0 &{} 0 \\ 0 &{} 0 &{} 0 &{}-\sqrt{f_h \mathcal {R}_{h,\zeta }} &{} 0 &{} 0 &{} 0 \\ 0 &{} 0 &{} 0 &{} 0 &{} \sqrt{\psi _v \mathcal {N}_{v,\zeta }} &{} 0 &{} 0 \\ 0 &{} 0 &{} 0 &{} 0 &{} -\sqrt{\lambda _{v,\zeta } \mathcal {S}_{v,\zeta }} &{} \sqrt{\lambda _{v,\zeta } \mathcal {S}_{v,\zeta }} &{} 0 \\ 0 &{} 0 &{} 0 &{} 0 &{} -\sqrt{f_v \mathcal {S}_{v,\zeta }} &{} 0 &{} 0 \\ 0 &{} 0 &{} 0 &{} 0 &{} 0 &{} -\sqrt{\nu _v \mathcal {E}_{v,\zeta }} &{} \sqrt{\nu _v \mathcal {E}_{v,\zeta }} \\ 0 &{} 0 &{} 0 &{} 0 &{} 0 &{} -\sqrt{f_v \mathcal {E}_{v,\zeta }} &{} 0 \\ 0 &{} 0 &{} 0 &{} 0 &{} 0 &{} 0 &{}-\sqrt{f_v \mathcal {I}_{v,\zeta }} \end{pmatrix} \sqrt{\Delta t} \end{aligned}$$

where ${{\varvec{I}}}_{15}$ is the 15 $\times 15$ identity.

The S-SEIRS model incorporates environmental perturbation and stochastic interactions among sub-populations of human hosts and vectors in different states. It provides a stochastic framework to study uncertainty and dynamics of malaria transmission as well as other mosquito-borne diseases.

### Model couplings

S-SEIRS model is coupled with the S-ELPAs formulation through the equality constraint of adult vector population. In essence, S-SEIRS represents different states (i.e. susceptible, exposed, infected) of adult vectors in S-ELPAs. Therefore, the total populations of adult mosquitoes in the two model are the same. The equality constraints are given as:

19a $$\begin{aligned} \mathcal {A}_{h,\zeta }(t) + \mathcal {A}_{r,\zeta }(t) + \mathcal {A}_{o,\zeta }(t)&= \mathcal {N}_{v,\zeta }(t) \end{aligned}$$

19b $$\begin{aligned} \mathcal {S}_{v,\zeta }(t) + \mathcal {E}_{v,\zeta }(t) + \mathcal {I}_{v,\zeta }(t)&= \mathcal {N}_{v,\zeta }(t) \end{aligned}$$

$\forall \zeta \in \mathbb {D}$ and $t > t_0$. For spatial movement of adult vectors among adjacent cells, it is assumed that vector populations are well-mixed or the fraction of malaria classes in the adult vector population remain unchanged during movements:

20a $$\begin{aligned} \frac{\mathcal {S}_{v,\zeta }(t_b)}{\mathcal {N}_{v,\zeta }(t_b)} \approx&\frac{\mathcal {S}_{v,\zeta }(t_a)}{\mathcal {N}_{v,\zeta }(t_a)}\end{aligned}$$

20b $$\begin{aligned} \frac{\mathcal {E}_{v,\zeta }(t_b)}{\mathcal {N}_{v,\zeta }(t_b)} \approx&\frac{\mathcal {E}_{v,\zeta }(t_a)}{\mathcal {N}_{v,\zeta }(t_a)} \end{aligned}$$

where $t_b$ and $t_a$ represent time before and after vector movement in every modelled time step $\Delta t$, respectively. Furthermore, birth and mortality rates of vectors in the S-SEIRS model are excluded as these processes are already represented in S-ELPAs model. The proposed coupling approach between S-ELPAs and S-SEIRS models presented allows to simulate stochastically the spatial dynamics of both vector population and malaria transmission over time. Finally, the coupled stochastic lattice-based vector dispersal and malaria model (SLIM) can be written as:

21a $$\begin{aligned} d\varvec{X}(t) =\,&\varvec{F}^x\big ( t,\varvec{X}(t),\varvec{p}^x(t, \varvec{C}^x(t) \big )dt \\&+ \varvec{G}^x\big (t,\varvec{X}(t), \varvec{p}^x(t, \varvec{C}^x(t) \big )d\varvec{W}(t)\end{aligned}$$

21b $$\begin{aligned} d\varvec{Y}(t) =\,&\varvec{F}^y\big ( t,\varvec{Y}(t),\varvec{p}^y(t, \varvec{C}^y(t) \big )dt \\&+ \varvec{G}^y\big (t,\varvec{Y}(t), \varvec{p}^y(t, \varvec{C}^y(t) \big )d\varvec{W}(t)\end{aligned}$$

21c $$\begin{aligned} \sum _{k=4}^{6} \varvec{X}_{k,\zeta }&(t) = \sum _{k=5}^{7} \varvec{Y}_{k,\zeta }(t), \quad \zeta \in \mathbb {D} \end{aligned}$$

in which ${{\varvec{X}}}(t)$ and ${{\varvec{Y}}}(t)$ are random variables described in (3) and (12). Solutions for equations (21) are obtained numerically.

### Estimating moisture index

The moisture index $\psi ^\mathsf {W}_{\zeta }$ shown in (1a) represents water availability in a particular cell $\zeta$ and is estimated using a sophisticated ecohydrologic modelling framework (Dhara, see [36]). The Dhara framework includes a collection of canopy process models (MLCan, see [37–39]) and a physically-based surface-subsurface flow model coupler (GCS-flow, see [40]) designed for capturing moisture transport on the land surface and in the below-ground systems. It incorporates vegetation acclimation to elevated CO$_2$ and the retention of moisture flow dynamics associated with topographic variability. This integration provides predictive capability to capture the impacts of environmental changes on the formation and persistence of breeding habitat. The SLIM (coupled S-ELPAs and S-SEIRS) model is linked with Dhara for incorporating climate-driven hydrologic factors to vector population and malaria transmission dynamics.

Female mosquitoes deposit eggs in breeding habitat of various sizes. However, a large fraction of the breeding habitat is at scales that are not detectable by currently available topographic data. As a result, there is always a probability that ponding exists in small-scale topographic depressions inside a particular non-saturated cell $\zeta$ that hydrologic modelling cannot capture. To address this scale mismatch issue, we incorporate the fractal structure found in topographic depressions to the estimation of $\psi ^\mathsf {W}_{\zeta }$. Specifically, the hypothesis is that topographic depressions exist at all sizes on the landscape following the power scaling law [41]. Available topographic data is used to find the scaling relationship of topographic depressions in bounded domain $\mathbb {D}$ and assume that this relationship remains unchanged at smaller scales for estimation of $\psi ^\mathsf {W}_{\zeta }$ as below:

22 $$\begin{aligned} \psi ^\mathsf {W}_{\zeta } = 10^{\alpha (1 - \Theta _{\zeta })} \end{aligned}$$

in which $\Theta _{\zeta } \in [\Theta _{min},1]$ is the degree of saturation of the soil surface in cell $\zeta$ obtained from the Dhara model, $\Theta _{min}$ is the minimum degree of saturation that is a function of soil properties, and $\alpha$ is the negative slope of the power-law scaling relationship for the exceedance probability of the area of topographic depressions found in the domain under consideration. In this work, $\alpha$ is identified separately using a topographic depression identification (TDI) algorithm [41].

## Model performance

In order to evaluate the performance of SLIM, each of its components (S-ELPAs and S-SEIRS) is first analysed independently. Then simulations of the fully coupled SLIM model using observed meteorological and topographic data for a case study in the rural area in Kilifi county, Kenya are presented.

### S-ELPAs model

S-ELPAs model simulations are implemented using similar parameter sets shown in a previous study [28] (Table 2). The model is tested in a simple rectangular domain $\mathbb {D}_1$ partitioned into uniform and lattice grid $\{\zeta := (i,j) \in \mathbb {Z}^2_{+} : i \le 5, j \le 5\}$. The size of each grid cell is $100\text{m} \times 100\text{m}$. In addition, periodic boundary conditions are applied. The model is run with four different initial conditions and sizes of vector population uniformly distributed over $\mathbb {D}_1$ to analyse the effects of random processes on mosquito population dynamics. As S-ELPAs is run independently, homogeneous moisture index and human distribution in $\mathbb {D}_1$ are assumed for simplicity. In addition, to isolate the effects of stochastic noise on the dynamics of vector population, all parameters in S-ELPAs are assumed to be constants over time. In other words, model parameters’ dependences on hydro-climatic factors are excluded in the simulations. S-ELPAs simulations are conducted in 800 days using daily time step.

**Fig. 4:**
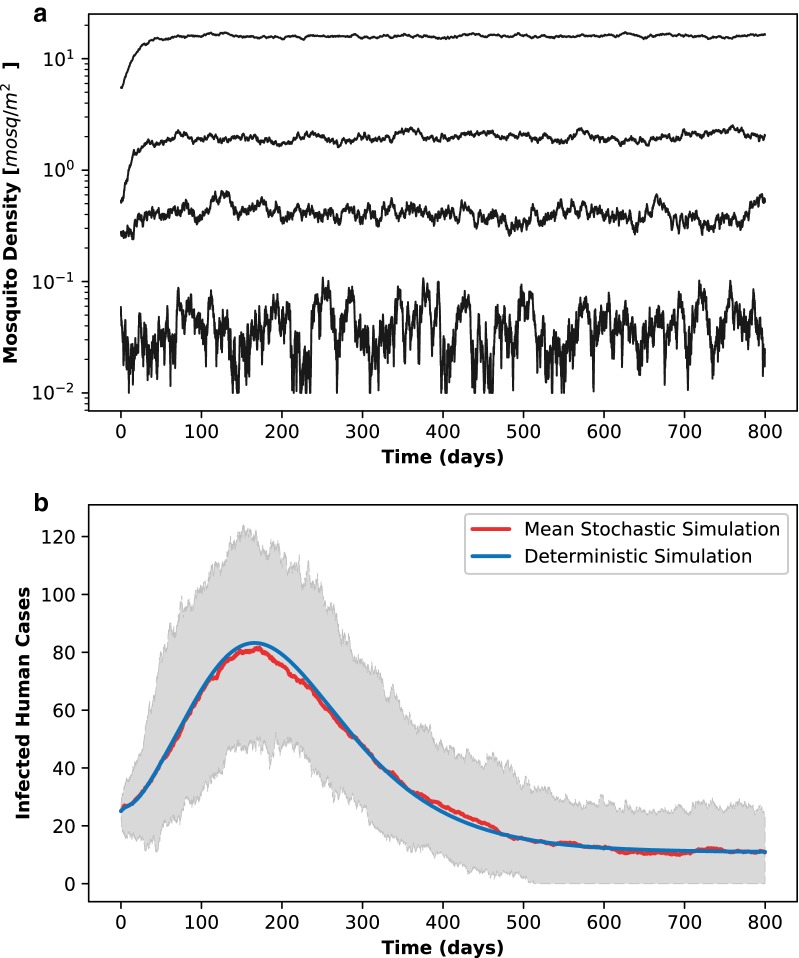
The dynamics of S-ELPAs and S-SEIRS models with white noise. **a** Simulation of total adult mosquitoes at different sizes of initial population. The graph shows how the oscillatory behavior becomes disrupted by noise in smaller populations, whereas large populations conform close to the equilibria. **b** Comparison of the malaria infected cases in humans between deterministic and stochastic simulations. The red curve shows the mean, and the gray shaded region shows the range for simulations of stochastic SEIRS model. The blue curve is from the original deterministic SEIRS model. While deterministic simulation tends to an endemic equilibrium, stochastic simulations show possible extinctions of the disease, as expected. The agreement between deterministic and mean stochastic simulations implies that a small fraction of the stochastic trajectory go to extinction in the simulations

Figure 4a shows the variations in log-scale of adult mosquito density averaged over $\mathbb {D}_1$ under the effects of random processes. It can be seen that S-ELPAs simulations with larger populations are affected slightly by stochastic noises, and the dynamics tend to be close to equilibria predicted by the deterministic systems. In contrast, smaller population sizes experience proportionally more noise and their behaviors tend to be further from the deterministic systems, highlighting the different dynamics of vector population at different sizes. The variations of small vector population can also be disrupted significantly by sudden changes induced by stochastic noises in the system. Linking these similarities and differences between stochastic and deterministic systems in meta-population and lattice-based models is thus important to study the dynamics of vector density in large areas, where populations at various sizes are interconnected.

### S-SEIRS model

A large number ($\sim$100) of independent S-SEIRS simulations are conducted and compared with the deterministic SEIRS model to investigate the modelled dynamics of malaria transmission in noise-dominated systems. All the stochastic and deterministic simulations have the same initial conditions chosen randomly. Moreover, periodic boundary conditions are applied for all simulations. Parameters for the two models are the same and selected from a previous published study [10]. Further, to separate the effects of stochastic noise on the dynamics of malaria, the dependences of parameters on hydro-climatic factors are also excluded in both S-SEIRS and SEIRS simulations. As the spatial movement of mosquitoes is not considered in S-SEIRS model, only simulations for a single grid cell are implemented. These simulations are conducted in 800 days with daily time step as well.

The variation of infected human malaria cases ($\mathcal {I}_h$) obtained from stochastic S-SEIRS (shaded area) and deterministic SEIRS (blue solid line) models are shown in Fig. 4b. Unlike the deterministic approach, S-SEIRS simulations provide a range of possibilities for malaria transmission given the same initial conditions and model parameters. S-SEIRS simulations shown in Fig. 4b highlight the random nature of malaria transmission that inevitably occur in real systems. Although the mean values of stochastic simulations (red solid line) are found to be close to those in the deterministic simulation, variability obtained from S-SEIRS implies that there are probabilities that (i) local outbreaks can be disrupted by random noises or stochastic extinctions may occur and (ii) the intensity of the outbreaks can be larger than the theoretical endemic equilibrium point shown in deterministic models. Note that periodic boundary conditions applied in the simulations imply isolated systems. This may allow shorter persistence time to extinction of malaria than in non-isolated systems. In our simple tests, the extinction of malaria in a specific region (i.e. entire domain) is defined as events in which the number of infected human population in this region is smaller than 0.5. Moreover, the probability of resurgence of the diseases in isolated systems is low. Capturing the range of variability and possible stochastic extinctions plays a fundamental role in understanding and breaking the circulation of the pathogen. This information, usually ignored in deterministic approaches, is important for preparing malaria control in reality.

### Case study

Next, the applicability of the SLIM model for large-scale simulations of malaria is demonstrated in a rural area in Kilifi county, Kenya. This region has high levels of malnutrition as well as a high incidence rate of *Plasmodium falciparum* parasites for which the *Anopheles gambiae* is the main vector [42]. Indeed, the intensity of malaria parasite transmission in Kilifi county is complex, subject to long- and short-term cycles of variation driven by climate, changes in human land use and the efficacy and coverage of interventions that target the parasite and vector [43]. Previous studies showed a decline in malaria transmission during the 1998–2009 period. However, there was a steady and marked increase of malaria transmission from 2009 to 2014 [43, 44]. Here, the primary objective is to present the capabilities and advantages of SLIM model for capturing the spatial and temporal variations of factors that drive malaria transmission. Therefore, the model is not validated for the case study. Model validation using observed malaria prevalence data and the impacts of climate change on malaria in coastal Kenya is presented in another work [45].

**Fig. 5:**
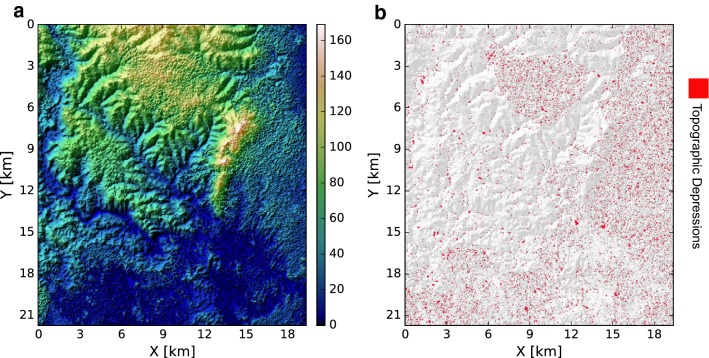
Domain of simulations in the case study at Kilifi county, Kenya. **a** Variation in topographic elevation. **b** Map of topographic depression (red polygons) identified from ASTER digital elevation model. The gray background represents hillshaded topography

The domain of simulation is approximately 440 km$^2$ (22 km north to south and 20 km east to west) with medium to high percentage cover of vegetation (Fig. 5). It is assumed that natural water on the ground surface is the main habitat of *Anopheles* mosquitoes. Topographic data at 30 m $\times$ 30 m resolution from Advanced Spaceborne Thermal Emission and Reflection radiometer (ASTER) global digital elevation model is used for modelling surface runoff and belowground soil moisture dynamics (Fig.  5a). Global reanalysis meteorological data at 3 h interval by European Centre for Medium-range Weather Forecasts (ECMWF) from 2005 to 2014 is used to drive both Dhara and SLIM models. Human population census data at 100 m spatial resolution is obtained from the population maps for low income nations [46]. Topographic depressions in the study area are found using TDI algorithm for estimating a moisture index as presented in "Estimating moisture index" (see [41]). The distributions of topographic depressions in the study domain are shown in Fig. 5b. The spatial heterogeneities of vector habitat and human hosts are the main drivers for mosquitoes movement and spread of malaria parasites. Model parameter sets similar to those used in previous studies [10, 28] are used for model simulations.

**Fig. 6:**
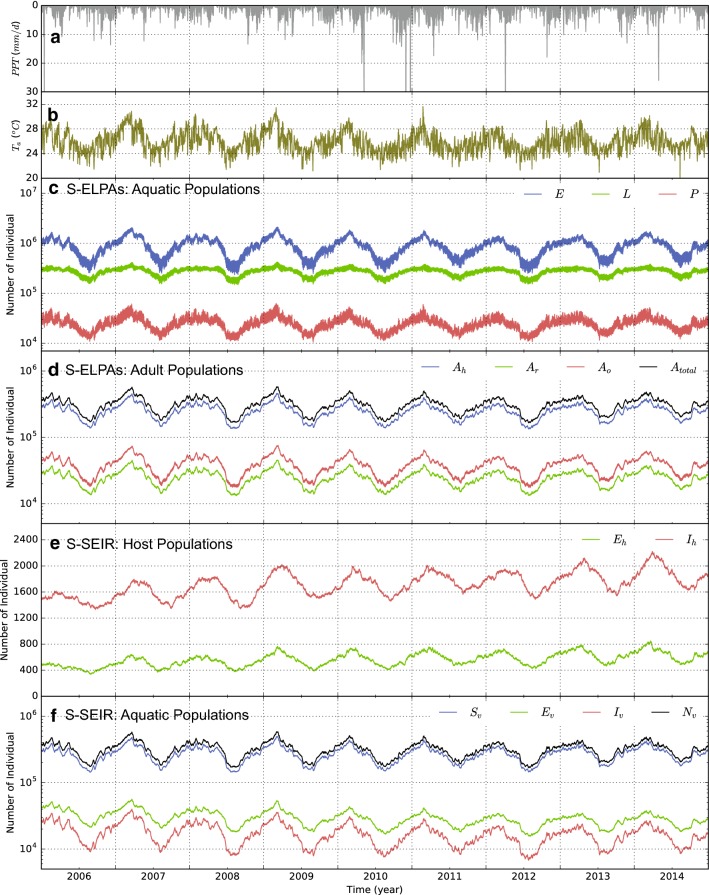
Illustration of variation of mosquitoes in different phases of their life cycle and malaria as predicted by SLIM model in response to meteorological driver for the study domain shown in Fig.  5. **a** Daily precipitation. **b** Mean daily air temperature. **c** Population dynamics of mosquitoes in aquatic phase; *E* represents egg population, *L* represents larvae population, and *P* represents pupae population, respectively. **d** Population dynamics of mosquitoes in adult phase; $A_h$ represents host-seeking mosquitoes, $A_r$ represents resting mosquitoes, and $A_o$ represents oviposition searching mosquitoes, respectively. **e** Dynamics of malaria within human hosts; $E_h$ represents exposed cases and $I_h$ represents infected cases, respectively. **f** Dynamics of malaria within human hosts; $S_v$ is susceptible vector, and $E_v$ represents exposed vector, $I_v$ is infected vector, and $N_v$ is the total vector, respectively.

Figure  6a–d presents the variation of total aquatic and adult phases of mosquito populations obtained from the S-ELPA component in SLIM. The results reveal that the variation of the mosquito population in both aquatic and adult stages are highly dependent on climatic factors. Specifically, positive correlations are found between monthly averaged mosquito populations modelled in S-ELPAs with observed monthly air temperature ($R^2 = 0.75 - 0.86$) and rainfall ($R^2 = 0.69 - 0.77$), respectively. The largest and smallest total mosquito population during the years are found correspondingly with the highest and lowest air temperature and rainy seasons with several days of time lag (10 and 18 days, respectively). In aquatic stages, the sensitivity of larvae development to air temperature change is found to be much lower than of eggs and pupae which was also shown in previous studies [29]. The population of adult *Anopheles* mosquitoes is also sensitive to climatic conditions (see Fig. 6d). The result shows that the fraction of host-seeking mosquitoes ($A_h$) in adult stage is high, consisting of $\sim 70-80\%$ of the total adult population. The sub-population of oviposition site searching mosquitoes ($A_o$) are usually $2-3$ times larger than the resting mosquitoes ($A_r$). The high number of egg deposited by female *Anopheles* during reproduction is likely a key factor that explains the high density of vectors in aquatic environment, thus mosquito population. The total number of adult mosquitoes is equal to the number of adults in the SEIRS model and plays a key role in malaria transmission.

The dynamics of malaria in human hosts and mosquito populations in the study area are presented in Fig.  6e–f. Positive correlations are also found between the monthly average malaria incidence ($\mathcal {E}_h$, $\mathcal {I}_h$) with air temperature ($R^2 = 0.58 - 0.69$) and rainfall ($R^2 = 0.53 - 0.67$), respectively. The results show that, similarly to the vector population, the variation of malaria incidences, including both exposed and infected cases, in the region is sensitive to climatic factors as it is directly dependent on vector density. The largest values of exposed human cases ($E_h$) are usually found after the rainy seasons start and air temperature was high. The peaks of $E_h$ are also followed by the largest values of infected human cases ($I_h$) in several days (Fig.  6e). During the peaks and troughs of the season, the rates of infected cases are about 2.5 and 1.0% of total population, respectively.

## Conclusions

In summary, we have presented a stochastic lattice-based integrated malaria (SLIM) model that consists of a vector dispersal (S-ELPAs) and a malaria dynamics (S-SEIRS) component. SLIM is developed to predict mosquito population dynamics and malaria transmission in response to heterogeneity and variability in the environment. It is well known that climatic and hydrological conditions strongly control *Anopheles* mosquito populations and thus influence malaria incidence, and indeed the associations have been demonstrated repeatedly. The details of malaria-environment interactions are highly nonlinear and uncertain both in time and space, thus the optimal predictive ability arises from complex models that involve processes from hydroclimatology, ecology, and entomology.

The stochastic coupled model is constructed based on deterministic systems [9, 10, 28]. The model is also link with a an ecohydrologic model (Dhara) used to capture soil moisture dynamics on the ground. This integration provides the capability to incorporate climate-driven hydrologic and ecologic processes with the dynamics of vector population and malaria transmission. In this manner, the presented SLIM model augments existing models by explicitly simulating all of the aforementioned complexities and incorporating a range of possible outcomes to the dynamics of vector population and transmission.

## References

[1] Miller LH, Baruch DI, Marsh K, Doumbo OK (2002). The pathogenic basis of malaria. Nature..

[2] Smith DL, McKenzie EF (2004). Statics and dynamics of malaria infection in Anopheles mosquitoes. Malar J..

[3] Anderson RM (1982). The population dynamics of infectious diseases: theory and applications. Population and community biology series.

[4] Anderson RM, May RM (1992). Infectious diseases of humans: dynamics and control. Dynamics and control.

[5] Paaijmans KP, Thomas MB (2011). Health: wealth versus warming. Nat Clim Change..

[6] Caminade C, Kovats S, Rocklov J, Tompkins AM, Morse AP, Colón-González FJ (2014). Impact of climate change on global malaria distribution. Proc Natl Acad Sci USA.

[7] Ross R. The prevention of malaria. 2nd ed. Dutton; 1910.

[8] MacDonald G. The Epidemiology and Control of Malaria. Oxford Medical Publications. Oxford, UK: Oxford University Press; 1957.

[9] Ngwa GA, Shu WS (2000). A mathematical model for endemic malaria with variable human and mosquito populations. Math Comput Model..

[10] Chitnis N, Cushing J, Hyman J (2006). Bifurcation analysis of a mathematical model for malaria transmission. SIAM J Appl Math..

[11] Yang HM (2000). Malaria transmission model for different levels of acquired immunity and temperature-dependent parameters (vector). Rev Saude Publica..

[12] Filipe JAN, Riley EM, Drakeley CJ, Sutherland CJ, Ghani AC (2007). Determination of the processes driving the acquisition of immunity to malaria using a mathematical transmission model. PLoS Comput Biol..

[13] Parham PE, Michael E (2010). Modeling the effects of weather and climate change on malaria transmission. Environ Health Perspect..

[14] Ariey F, Duchemin JB, Robert V (2003). Metapopulation concepts applied to falciparum malaria and their impacts on the emergence and spread of chloroquine resistance. Infect Genet Evol..

[15] Bomblies A, Duchemin JB, Eltahir EAB (2008). Hydrology of malaria: model development and application to a Sahelian village. Water Resour Res..

[16] Gu W, Novak RJ (2009). Agent-based modelling of mosquito foraging behaviour for malaria control. Trans R Soc Trop Med Hyg..

[17] Arifin SN, Zhou Y, Davis GJ, Gentile JE, Madey GR, Collins FH (2014). An agent-based model of the population dynamics of Anopheles gambiae. Malar J..

[18] Pizzitutti F, Pan W, Barbieri A, Miranda JJ, Feingold B, Guedes GR (2015). A validated agent-based model to study the spatial and temporal heterogeneities of malaria incidence in the rainforest environment. Malar J..

[19] Mandal S, Sarkar R, Sinha S (2011). Mathematical models of malaria - a review. Malar J..

[20] Reiner RC, Perkins TA, Barker CM (2013). A systematic review of mathematical models of mosquito-borne pathogen transmission: 1970–2010. J R Soc Interface..

[21] Smith DL, Perkins TA, Reiner RC, Barker CM, Niu T, Chaves LF (2014). Recasting the theory of mosquito-borne pathogen transmission dynamics and control. Trans R Soc Trop Med Hyg..

[22] Keeling MJ, Rohani P (2008). Modeling infectious diseases in humans and animals.

[23] Azaele S, Maritan A, Bertuzzo E, Rodriguez-Iturbe I, Rinaldo A (2010). Stochastic dynamics of cholera epidemics. Phys Rev E..

[24] Herwaarden OA, Grasman J (1995). Stochastic epidemics: major outbreaks and the duration of the endemic period. J Math Biol..

[25] van Herwaarden AO (1997). Stochastic epidemics: the probability of extinction of an infectious disease at the end of a major outbreak. J Math Biol..

[26] Britton T (2010). Stochastic epidemic models: a survey. Math Biosci..

[27] Krstic M (2011). The effect of stochastic perturbation on a nonlinear delay malaria epidemic model. Math Comput Simul..

[28] Lutambi AM, Penny MA, Smith T, Chitnis N (2013). Mathematical modelling of mosquito dispersal in a heterogeneous environment. Math Biosci..

[29] Depinay JM, Mbogo C, Killeen G, Knols B, Beier J, Carlson J (2004). A simulation model of African Anopheles ecology and population dynamics for the analysis of malaria transmission. Malar J..

[30] Allen E. Modeling with Itô Stochastic differential equations. Mathematical modelling: theory and applications. Heidelberg, Germany: Springer Berlin Heidelberg; 2007.

[31] Allen LJS (2010). An introduction to stochastic processes with applications to biology.

[32] Doolan DL, Dobaño C, Baird JK (2009). Acquired immunity to malaria. Clin Microbiol Rev..

[33] Detinova TS (1962). Age-grouping methods in Diptera of medical importance with special reference to some vectors of malaria. WHO Monograph series..

[34] Briere JF, Pracros P, Le Roux AY, Pierre JS (1999). A novel rate model of temperature-dependent development for arthropods. Environ Entomol..

[35] Paaijmans KP, Read AF, Thomas MB (2009). Understanding the link between malaria risk and climate. Proc Natl Acad Sci USA.

[36] Le PVV, Kumar P (2017). Interaction between ecohydrologic dynamics and microtopographic variability under climate change. Water Resour Res..

[37] Drewry DT, Kumar P, Long S, Bernacchi C, Liang XZ, Sivapalan M (2010). Ecohydrological responses of dense canopies to environmental variability: 1. Interplay between vertical structure and photosynthetic pathway. J Geophys Res..

[38] Le PVV, Kumar P, Drewry DT, Quijano JC (2012). A graphical user interface for numerical modeling of acclimation responses of vegetation to climate change. Comput Geosci..

[39] Le PVV, Kumar P, Drewry DT (2011). Implications for the hydrologic cycle under climate change due to the expansion of bioenergy crops in the Midwestern United States. Proc Natl Acad Sci USA.

[40] Le PVV, Kumar P, Valocchi AJ, Dang HV (2015). GPU-based high-performance computing for integrated surface-sub-surface flow modeling. Environ Modell Softw..

[41] Le PVV, Kumar P (2014). Power law scaling of topographic depressions and their hydrologic connectivity. Geophys Res Lett..

[42] Nyakeriga AM, Troye-Blomberg M, Chemtai AK, Marsh K, Williams TN (2004). Malaria and nutritional status in children living on the coast of Kenya. Am J Clin Nutr..

[43] Snow RW, Kibuchi E, Karuri SW, Sang G, Gitonga CW, Mwandawiro C (2015). Changing malaria prevalence on the Kenyan Coast since 1974: climate, drugs and vector control. PLoS ONE..

[44] Mogeni P, Williams TN, Fegan G, Nyundo C, Bauni E, Mwai K (2016). Age, spatial, and temporal variations in hospital admissions with malaria in Kilifi County, Kenya: a 25-year longitudinal observational study. PLoS Med..

[45] Le PVV, Kumar P, Ruiz MO, Mbogo C, Muturi JE. Predicting the direct and indirect impacts of climate change on malaria in coastal Kenya. PLOS (under review). 2018. 10.1371/journal.pone.0211258 PMC6364917 30726279

[46] Tatem AJ, Noor AM, von Hagen C, Di Gregorio A, Hay SI (2007). High resolution population maps for low income nations: combining land cover and census in East Africa. PLoS ONE..

